# Analgesic Management of Chronic Pancreatitis

**DOI:** 10.1007/s10620-025-09626-3

**Published:** 2025-12-24

**Authors:** Dominic Amakye, Hadie Razjouyan, Matthew D. Coates

**Affiliations:** 1 Division of Gastroenterology, Department of Medicine, University of Tennessee Health Science Center, Memphis, TN USA; 2 Division of Gastroenterology & Hepatology, Department of Medicine, Penn State College of Medicine, Hershey, PA USA; 3 Department of Neuroscience and Experimental Therapeutics, Penn State College of Medicine, Hershey, PA USA

**Keywords:** Analgesics, Chronic pancreatiti, Abdominal pain, Pain management

## Abstract

Abdominal pain is the most prevalent symptom of chronic pancreatitis and, when inadequately controlled, it leads to impaired quality of life across physical, psychological, and social domains, along with increased healthcare utilization and reduced life expectancy. Effective pain control remains challenging due to the multifactorial and complex etiology of pain, yet it is essential for improving patient outcomes.

An accurate pain assessment using validated clinical tools is a critical first step toward optimizing management. Effective treatment often requires a multidisciplinary, multimodal approach that integrates lifestyle modifications, including smoking and alcohol cessation, with stepwise pharmacologic therapy. For patients with refractory pain, surgical and endoscopic interventions are viable treatment options.

This review provides a comprehensive overview of non-pharmacologic, pharmacologic, endoscopic, and surgical strategies for managing painful chronic pancreatitis, emphasizing evidence-based approaches that improve patient outcomes.

## Epidemiology, Mechanisms and Impact of Pain in Chronic Pancreatitis

Abdominal pain is the most prevalent symptom associated with chronic pancreatitis (CP), occurring in about 85% of patients [1–3]. The exact etiology of CP-related pain is not well understood, but it is often multifactorial. The intense inflammatory cascade associated with pancreatitis triggers the release of cytokines and other signaling molecules that activate several membrane receptors, stimulating the transmission of nociceptive signals to pain centers of the brain [4]. Additionally, pancreatic ductal obstruction from stones or strictures leads to increased intraluminal pressures above the physiologically tolerable levels, contributing to pain [5]. Recurrent pancreatic inflammation may also damage pancreatic innervation, leading to neuronal remodeling, hypersensitivity, and neuropathic pain syndromes, including hyperalgesia and allodynia, even in the absence of ongoing nociceptive input [6]. These peripheral changes can amplify central sensitization and alter pain perception [4, 7].

The clinical presentation of CP-related abdominal pain is heterogeneous. Patients may experience intermittent acute pain, chronic pain of fluctuating intensity, or persistent baseline pain punctuated by acute exacerbations [8]. A study by Mullady et al. classified CP-related pain into five distinct categories; *type A*, intermittent episodes with pain-free intervals; *type B*, prolonged periods of daily pain; *type C*, predominantly pain-free with occasional severe episodes; *type D*, constant mild pain interspersed with severe episodes; and *type E*, unremitting severe pain [8]. Typically pain is epigastric, may radiate to the back, and is often precipitated or exacerbated by meals [9]. Notably, the pain phenotype does not always correlate with the underlying pathophysiological mechanisms [10]. Although pain is the defining feature of CP, 10–15% of patients, interestingly, remain asymptomatic and are classified as painless CP [3, 11, 12].

Management of CP-related pain is often difficult, as no single intervention reliably provides complete relief [8]. Patients with inadequately controlled pain are more likely to undergo multiple procedures with increased risk of complications and morbidity, which leads to a decreased life expectancy [13]. Pain also drives greater healthcare utilization, imposes substantial economic costs, and profoundly diminishes quality of life across physical, social, and psychological domains [14–16]. For instance, a systematic review evaluating the prevalence of depression in chronic pancreatitis in 87,136 patients reported that approximately 1 in 3 patients has depression [17]. Another study compared outcomes of patients with constant pain patterns to patients with intermittent pain patterns; patients with constant pain, regardless of severity, had higher rates analgesia use, hospitalization and disability [18]. The economic burden of CP and the abdominal pain associated with this disorder are substantial. For example, in the United States alone, healthcare expenditures related to this condition may exceed $2 billion annually [19].

Given its prevalence, complexity, and profound impact on patients and health systems, effective management of CP-related pain is essential. This review offers a comprehensive guide to the multifaceted strategies available for optimal pain control, aimed at guiding individualized, multidisciplinary approaches to improve outcomes in painful CP.

### Pain Assessment in Chronic Pancreatitis

An accurate evaluation of pain in CP is essential for effective management. Given the multifactorial nature of CP-related pain, assessment tools must capture its biological, psychological, and social dimensions. Commonly used pain rating scales, such as the visual analog scale and numeric rating scale, primarily measure pain intensity but fail to account for pain frequency or qualitative characteristics, limiting their utility in the context of CP.[7, 20].

More comprehensive instruments, such as the brief pain inventory assessment scale, offer validated self-administered questionnaires that assess both pain severity and its impact on daily activities and mood, and are suitable for use in CP [17]. Pancreatic quantitative sensory testing is another useful tool that stratifies patients into three distinct phenotypes, including widespread hyperalgesia, segmental hyperalgesia, and no hyperalgesia, which are independent of any underlying psychiatric condition [2]. The McGill Pain Questionnaire also contributes to CP pain assessment by providing a multidimensional evaluation. It provides a quantitative measure that incorporates the affective, qualitative, and sensory nature of the pain, thereby enhancing the understanding of pain experiences [21]. The Comprehensive Pain Assessment Tool (COMPAT) provides a detailed evaluation of chronic pancreatitis–related pain. [22]. However, it includes multiple questions and requires more than 30 min to complete, which limits its practicality in routine clinical settings. [23]. A shorter version, the COMPAT-SF, assesses five pain dimensions using six questions. It has been validated, demonstrating strong correlation with the Brief Pain Inventory (*p* < 0.0001), and has been translated into multiple languages to facilitate wider use. [23]. It, however, does not assess the psychosocial aspects of CP-related pain.

## Analgesic Management

The management of CP-associated abdominal pain often requires a multimodal approach, incorporating both pharmacological and non-pharmacological interventions.

### Non-invasive, Non-pharmacological Strategies

Lifestyle changes and dietary modifications play a crucial role in enhancing the quality of life and reducing pain in CP patients. Numerous studies have consistently demonstrated that smoking and alcohol use exacerbate painful relapses [24, 25]. Notably, smoking cessation has been shown to sustain the long-term benefits of endoscopic therapy for CP-related pain [26]. Therefore, cessation of smoking and alcohol use is strongly recommended.

In the early stages of CP, pain can result from an increase in pancreatic ductal pressure triggered by stimulation of the exocrine pancreas via cholecystokinin (CCK) following a fatty meal [28] Dietary strategies such as a low-fat elemental diet with purified amino acids have been shown to reduce pain and enhance nutritional status in individuals with CP [27]. Medium-chain triglycerides (MCT) also suppress CCK secretion, leading to decreased pain levels [28]. A pilot study revealed a significant improvement in pain scores over 10 weeks in patients following a diet rich in MCTs [29]. Similarly, a study involving 596 patients reported a significant pain reduction after 12 weeks on a low-fat elemental diet [30]. These dietary interventions appear most effective in the early stages of CP, when exocrine pancreatic function is still preserved, compared to the late stages, where pain is predominantly neurally mediated [29].

Behavioral interventions are also recommended as part of a multidisciplinary approach to CP-related pain management. The International Consensus Guidelines advocate for their incorporation, particularly in patients experiencing psychological distress [1]. Among the various behavioral interventions, cognitive behavioral therapy (CBT) and biofeedback are effective in reducing chronic abdominal pain [31]. A recent RCT evaluating the effectiveness of CBT in 30 patients with CP-related pain demonstrated a reduction in pain intensity and interference at 3 months in the CBT group compared to controls. The control received usual care, which included regular office visits and multimodal pain management. [16].

Pancreatic enzyme replacement therapy may alleviate abdominal pain by reducing CCK secretion and pancreatic stimulation through protease-mediated denaturation of CCK-releasing peptide in the duodenum [28]. Non-enteric-coated formulations, designed for duodenal release, were hypothesized to be especially effective [32]. However, a meta-analysis performed by *Yaghoobi *et al*.* found no difference in analgesic consumption or daily pain scores between the pancreatic enzyme supplementation and placebo, casting doubt on its analgesic efficacy [33]. Differences in the efficacy of enzyme replacements may be due to differences in medication formulations. In the U.S., most pancreatic enzymes are enterically coated to facilitate release in the mid-small bowel, and this may be ineffective in suppressing the CCK release, which contributes to pain. Additionally, differences in study subject characteristics may be contributory since the enzymes were most effective in patients who had undergone surgery on the pancreas [33]

CP-related pain is often linked to micronutrient deficiencies from malabsorption, which predispose to oxidative stress, inflammation, and pancreatic injury [34, 35]. Antioxidant supplementation has been explored as a therapeutic option. A 2015 meta-analysis reported pain reduction with methionine-containing antioxidants combined with selenium, ascorbate, beta carotene, and alpha tocopherol over 10–12 months, though adverse effects such as headaches, constipation, and heartburn were common [36]. Conversely, a long-term study of 30 CP spanning over 10 years found no significant benefit [37]. The American College of Gastroenterology (ACG) conditionally recommends antioxidant therapy for pain relief, particularly in the early stages of disease [38].

### Non-invasive Pharmacologic Strategies

Currently, there are no definitive guidelines for the optimal analgesic strategy in managing abdominal pain associated in CP. The WHO three-step analgesic ladder originally developed for cancer pain and chronic pain management, is frequently applied to CP [39, 40]. This framework recommends initiating treatment with non-opioid medications and escalating doses before introducing opioids [41].

Acetaminophen is considered a first-line agent due to its favorable safety profile [1]; however, its efficacy in CP-related pain has not been extensively studied, and it is generally not recommended as monotherapy due to limited effectiveness [7, 28]. NSAIDS are the next line of treatment, though their use is constrained by gastrointestinal (GI) adverse effects, including peptic ulcers and GI bleeding [28]. Among NSAIDS, ibuprofen, diclofenac, and celecoxib are associated with a lower risk of serious GI complications [42]. When prescribed, NSAIDs should be used for short duration and long-acting formulations should be avoided. In patients with elevated bleeding risk, co-administration of proton pump inhibitors is advised [43].

Pain in CP is influenced by alterations in both the peripheral and central nervous systems, including increased cerebral cortical thickness and pancreatic nerve fiber density, which contribute to pain amplification over time [4]. Centrally acting agents such as pregabalin and gabapentin, commonly used for neuropathic pain, may offer benefit in CP-related pain [7]. Pregabalin has demonstrated potential as an adjuvant analgesic. In a study by Olesen et al., escalating doses of pregabalin over three weeks significantly improved abdominal pain in CP patients compared to placebo (36% vs 24%, *p* = 0.02) [44]. Adverse effects of gabapentinoids, including dizziness, drowsiness, and lightheadedness, are common, particularly when combined with opioids [44]. Patient education regarding these risks is recommended before initiating therapy. Although SSRIs, amitriptyline, and SNRIs, are effective for neuropathic pain, they have not demonstrated efficacy in CP pain management [1, 44].

Opioids may be considered for persistent CP-related pain that is unresponsive to first-line analgesics, in accordance with the WHO pain ladder. However, long-term use is generally discouraged, due to risks of dependence, abuse, tolerance, and adverse effects, especially in individuals with a history of alcohol use [38, 45]. Despite these concerns, opioid use for CP-related pain remains high in the USA [46]. A recent study involving 681 CP patients found that 44% regularly used strong opioids, which was associated with reduced quality of life, increased healthcare utilization, and greater disability [45]. Pain patterns and prior celiac plexus block were identified as independent predictors of higher opioid dose and frequency.

**Fig. 1:**
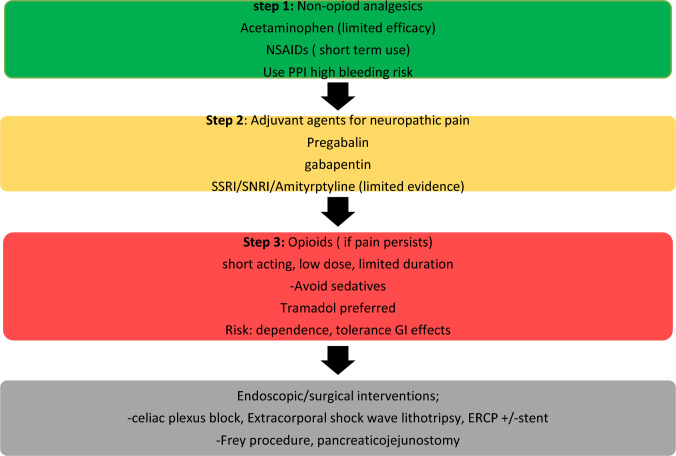
Stepwise algorithm to management of chronic pancreatitis-related pain

When opioids are necessary, short-acting formulations at the lowest effective dose for a limited duration are preferred [47]. A study by *Wilder-Smith *et al., involving 25 CP patients, validates this approach, showing that tramadol provided superior pain control compared to morphine after four days, with 67% of patients reporting effective analgesia versus 20% in the morphine group [48]. Tramadol also exhibited a favorable GI profile, with no change in orocecal transit time, whereas morphine increased both orocecal and colonic transit times. [48] Concurrent use of opioids with other sedating medications, such as benzodiazepines, should be avoided if possible [49]. Figure 1 outlines a stepwise process of analgesia use in CP.

### Minimally Invasive and Surgical Strategies

For patients with refractory pain despite optimal medical management, minimally invasive therapies or surgery may be necessary.

Celiac plexus block has been evaluated as a treatment for CP-related pain. This treatment modality involves the injection of bupivacaine with or without triamcinolone into the celiac plexus via endoscopic ultrasound (EUS) or under CT guidance [50]. A study by Gress et al., EUS-guided celiac plexus block led to improved pain scores in 55% of participants. Mean pain scores decreased from 8 to 2 at 4 and 8week follow-ups, with a small subset maintaining analgesic benefit at 48 weeks [51]. A more recently published systematic review also showed the effectiveness of EUS-guided celiac plexus block, with 64% of 621 patients having adequate pain control. The median duration of analgesia, however, varied from 27 days to 8 months [52]. CT-guided block is an alternative approach; however, studies suggest that EUS-guided blocks offer greater patient satisfaction, improved efficacy, and longer-lasting pain relief. In one study of 22 patients, 40% of those receiving EUS-guided blocks reported ongoing analgesic benefit at 8 weeks, compared to 25% in the CT-guided group [50]. Reported complications from celiac plexus blockade are low and include diarrhea, pain exacerbation, hypotension, gastric hematoma, and bleeding [52–54]. However, one study of 220 patients reported a serious complication rate of 1.8 [53].

Endoscopic procedures aimed at decompression of the pancreatic duct and improving ductal flow are effective in patients with a dilated pancreatic duct [4, 48, 49]. *Ponchon *et al. evaluated pancreatic duct stenting in 23 CP patients and found that 74% discontinued analgesics at 6 months, and 52% at 12 months. Pain relief was associated with reduced ductal diameter and resolution of strictures [55]. Extracorporeal shock wave lithotripsy (ESWL) can be added to endoscopic procedures to fragment and remove large stones [56]. An RCT comparing ESWL alone to ESWL combined with endoscopic drainage (ERCP) in 55 patients with chronic calcified pancreatitis found a similar reduction in pain episodes over the first year—mean decreases of 3.8 and 3.7, respectively—with no significant difference between groups (*p *= 0.759). However, treatment costs were three times higher in the combined group [57].

Surgery plays a crucial role in CP-related pain management. The benefits of surgery have been demonstrated in multiple studies; a randomized control trial assessing the effects of early surgery vs endoscopy first approach for pain in CP showed improved pain scores in the surgery group compared to endoscopy (37 vs 49; between-group difference, − 12 points [95% CI, − 22 to − 2]; *p* = 0.02). Surgical drainage procedures included lateral pancreaticojejunostomy for pancreatic head lesions < 4 cm and duodenal preserving pancreatic head resection for an enlarged pancreatic head > 4 cm (Frey procedure) [58] Additionally, early surgical intervention (within three years of disease onset) yields better outcomes than delayed surgery [59]. Several surgical options have been described, including decompression procedures, pancreatic resection, and denervation. Current guidelines from the American Society for the Study of Gastrointestinal Endoscopy (ASGE) recommend surgical intervention over endoscopic management in patients with painful CP and an obstructed pancreatic duct, provided no surgical contraindications exist [60]. Nevertheless, many patients and providers opt to begin with less invasive endoscopic approaches before considering surgery [60]. In patients who are not candidates for endoscopic therapy or extracorporeal shock wave lithotripsy (ESWL)—particularly when anatomical factors such as stone or stricture location in the body or tail of the pancreas are present—surgery should be the preferred first-line approach [61]. Table 1 summarizes the different endoscopic and surgical therapies for CP-related pain; the benefits and risk associated with each procedure.

**Table 1:** Benefits and risks of procedures for CP-related pain

Procedure	Benefits	Risks / limitations
Celiac plexus block (EUS or CT-guided)	Provides pain relief in ~ 55–64% of patients Minimally invasive option for refractory pain	Relief often temporary (27 days–8 months Complications: diarrhea, pain flare, hypotension, gastric hematoma, bleeding Serious complication rate ~ 1.8%
Endoscopic therapy (pancreatic duct stenting, ERCP)	Effective in patients with dilated pancreatic duct Associated with ductal diameter reduction and stricture resolution	Requires repeat procedures Limited to ductal obstruction cases Risks: infection, bleeding, pancreatitis
Extracorporeal shock wave lithotripsy (ESWL)	Breaks down large stones for removal RCTs show similar pain reduction whether used alone or with ERCP	May need multiple sessions Effectiveness depends on stone size/location
Surgery (drainage, resection, frey procedure, denervation)	Provides more durable pain relief than endoscopy Early surgery (< 3 years from onset) yields better outcomes Effective for obstructed ducts and complex anatomy	Higher morbidity and longer recovery Risks: infection, fistula, bleeding Requires careful patient selection; contraindications may limit use

## Conclusion

Effective management of CP-related pain is challenging and requires accurate assessment with validated clinical tools to guide therapeutic decisions. Lifestyle modifications, particularly smoking and alcohol cessation, are important components of treatment. Comprehensive, multidisciplinary care involving gastroenterologists, dietitians, psychiatrists, and primary care providers is essential, not only for optimizing symptom control but also for addressing comorbidities such as addiction, depression, and anxiety, thereby reducing the overall healthcare burden of CP [62].

A stepwise approach to analgesia is recommended, beginning with non-opioid agents, progressing to gabapentinoids, and reserving opioids for refractory cases. For patients with pain refractory to medical therapy, surgery is considered the first-line intervention, especially in the setting of an obstructed pancreatic duct. Nevertheless, many patients and providers favor less invasive strategies; endoscopic interventions or ESWL may be pursued following multidisciplinary discussions, with surgical options carefully weighed. This patient-centered, stepwise strategy remains the cornerstone of effective CP-related pain management.

## Data Availability

No datasets were generated or analysed during the current study.
